# Catalytic Degradation of Diatrizoate by Persulfate Activation with Peanut Shell Biochar-Supported Nano Zero-Valent Iron in Aqueous Solution

**DOI:** 10.3390/ijerph15091937

**Published:** 2018-09-06

**Authors:** Jian Xu, Xueliang Zhang, Cheng Sun, Huan He, Yuxuan Dai, Shaogui Yang, Yusuo Lin, Xinhua Zhan, Qun Li, Yan Zhou

**Affiliations:** 1 State Key Laboratory of Pollution Control and Resource Reuse, School of the Environment, Nanjing University, Nanjing 210023, China; xujian@nies.org (J.X.); daiyuxuan@smail.nju.edu.cn (Y.D.); 2 Nanjing Institute of Environmental Sciences, Ministry of Environmental Protection, Nanjing 210042, China; 2016103039@njau.edu.cn (X.Z.); lys@nies.org (Y.L.); liqun@nies.org (Q.L.); zhouyan@nies.org (Y.Z.); 3 College of Resources and Environmental Sciences, Nanjing Agricultural University, Nanjing 210095, China; xhzhan@njau.edu.cn; 4 School of Environment, Nanjing Normal University, Nanjing 210023, China; huanhe@njnu.edu.cn

**Keywords:** nano zero-valent iron, peanut shell biochar, persulfate activated, diatrizoate, degradation

## Abstract

An emerging pollutant, diatrizoate (DTZ) has been frequently detected in aqueous solution. Unique reticular peanut shell biochar (BC)-supported nano zero-valent iron (nZVI) composite (nZVI/BC) was successfully synthesized and used as a catalyst for activating persulfate (PS) to promote the removal of DTZ. The structure and morphology of the nanocomposite materials were characterized by scanning electron microscopy, X-ray diffraction, Brunauer-Emmett-Teller measurements, and Fourier transform infrared spectroscopy. The degradation of DTZ (20 mg L^−1^) was achieved by activating PS with the nanocomposite material. The removal of DTZ reached nearly 100% using 25 mM PS and 0.45 g L^−1^ nZVI/2BC (mass ratio of nZVI and BC at 1:2) nanocomposite material at pH 3.0 and 25 °C. Influencing factors, such as dosages of nZVI/2BC and PS, temperature, and pH were also investigated. The mechanisms of PS activation with nZVI/2BC were discussed, including BC property, electron transfer, and the identification of free radicals in the reaction. The findings demonstrated that nZVI/BC-PS (peanut shell BC-supported nZVI activating PS) is a promising material for the treatment of refractory organic pollutants.

## 1. Introduction

With the rapid development of medicine, considerable attention is being paid to pharmaceuticals and personal care products (PPCPs), which are unfortunately also emerging contaminants (ECs) [1,2]. Iodinated contrast media (ICM) is a typical PPCP, which is used to enhance organs and blood vessel image observation effects [3]. ICM was widely detected in hospital and domestic wastewater, treated wastewater effluent, groundwater, surface water, and even drinking water, ranging from ng L^−1^ to μg L^−1^ worldwide due to its abuse and improper disposal [4,5]. China is one of the largest consumers of ICM in the world, with total ICM concentrations in Taihu Lake and the Huangpu River ranging from 88.7 to 131 ng L^−1^ and 102 to 252 ng L^−1^, respectively [6]. Diatrizoate (DTZ), an abundant ionic ICM, is identified as a refractory organic pollutant due to it being highly polar and chemically inert [7,8], which exceeds the self-purification capacity of the environment and hence may result in potential hazards to living species in various ways. For example, previous studies reported that DTZ led to nephrotoxic effects and decreased thyroid homeostasis of organisms [9,10]. Therefore, it is necessary to find out efficient treatment technologies to remove DTZ from the aquatic environment. However, there have been few reports on DTZ degradation so far.

Advanced oxidation processes (AOPs) based on hydroxyl radical (HO^•^) (E_0_ = 1.8–2.7 V) have been recognized as efficient techniques for the remediation of recalcitrant organic pollutants [11], while AOPs based on sulfate radical (SO_4_^•−^) have stronger oxidation ability (E_0_ = 2.5–3.1 V). Compared with hydroxyl radical (HO^•^), sulfate radical has longer life, and sulfate radical is also more selective than hydroxyl radical [11]. Many researchers have made efforts to produce SO_4_^•−^ by activating persulfate (PS) for the removal and mineralization of refractory contaminants. Up to now, heat, UV light, transition metals, ultrasound, and alkalization have been considered to be the main activation methods [12,13].

Among the above-mentioned activation methods, Fe, a cheap and non-toxic transition metal, is considered as an efficient material in activating PS. Compared to Fe^3+^ and Fe^2+^, which have low efficiency in practice [14,15], zero-valent iron (ZVI) is widely known as an environmentally friendly material and can act as an alternative source of Fe^2+^ by continuous electron transfer [15]. Many studies have been carried out using ZVI to activate PS so as to effectively produce SO_4_^•−^ for removing organic pollutants, including 2,4-dinitrotoluene [16], p-chloroaniline [17], Acid Orange 7 [18], trichloroethylene [19], and so forth. This is because ZVI (especially nano-ZVI) has a small particle size, large specific surface area, and strong reactivity [20]. However, due to its high surface energy and intrinsic magnetic interactions, nano-ZVI (nZVI) is easy to aggregate into microscale particles, which decreases the property of its activation [21]. Various strategies have been used to try and disperse nanomaterials, such as by using surface coating and green synthesis methods [22,23], and using supporting materials such as graphene, resin, active carbon, and zeolite [24,25,26,27].

Biochar (BC) is a rich and inexpensive material, made in an anaerobic environment by combusting biomass [28,29]. Biochar has been proposed as an environmentally friendly remedial material for a variety of environmental applications [30,31]. Through pyrolysis, a porous structure is formed in BC, which can be used to disperse and stabilize nZVI [32,33]. Abundant oxygen-containing functional groups, including hydroxyl (-OH) and carboxyl (-COOH), can also be observed on the surface of BC. These functional groups are able to activate PS, along with nZVI [34,35]. In addition, BC has a large surface area and excellent ion exchange capacity, and hence can adsorb many heavy metal ions and organic contaminants. Adsorptions of Reactive Black 5 dye, Cu^2+^, Pb^2+^, and Cr^3+^ have been reported by peanut shell BC in recent years [36,37,38]. The mesh structure of peanut shell BC may be easier to disperse the nZVI particle, while other commonly porous BCs are prone to blocking [1,33,39], and nZVI supported on peanut shell BC composite material (nZVI/BC) with a mesh structure should have excellent degradation ability. The development of biological waste-based innovative materials for environmental remediation could greatly help conquest widespread environmental pollution problems in developing countries like China [40,41], while rendering relatively low economic cost and environmental footprints [42,43]. However, up to now, the study of nZVI supported on peanut shell BC composite material with a mesh structure has been rarely reported.

The aims of this work are: (1) to prepare and characterize BC and nZVI/BC composites; (2) to evaluate the synergetic effects between Fe and BC on activation PS, and the activation ability of PS on the degradation of DTZ with nZVI/BC composite in aqueous solution; (3) to recognize the significant influencing factors that affect its activation ability and optimize the conditions in DTZ removal; (4) to explore the PS activation mechanism by nZVI/BC composites.

## 2. Materials and Methods

### 2.1. Materials

Sodium diatrizoate hydrate (C_11_H_8_I_3_N_2_NaO_4_·xH_2_O, CAS 737-31-5, >98%), sodium borohydride (NaBH_4_, >98%), iron (II) sulfate heptahydrate (FeSO_4_·7H_2_O, >99%), and sodium persulfate (Na_2_S_2_O_8_, >99%) were provided by the Sigma company in China. Only high-purity analytical-grade chemicals and deionized water (18.25 MΩ·cm) was used in this study.

### 2.2. Experiments

#### 2.2.1. Synthesis Process of BC and nZVI/BC

The raw material peanut shells were collected from Nanjing, Jiangsu province, China. The obtained materials were washed by deionized water several times to remove impurities and then dried in an oven at 70 °C until a constant weight was achieved. After grinding by a ball mill and passing through a 100-mesh sieve, the materials were pyrolyzed in a muffle furnace under an oxygen-limited condition at 350 °C for 2 h. The cooling-off BC was treated with 1 mol L^−1^ HCl for 12 h and washed three times with deionized water.

The nZVI/BC was prepared by reducing ferrous iron (FeSO_4_·7H_2_O) with sodium borohydride (NaBN_4_) [44]. To take nZVI/BC mass ratios of 1:1 as an example, 5.0 g FeSO_4_·7H_2_O and 1.0 g BC were put in a 3 mouth-flask with 250 mL deionized water and stirred by a mechanical agitator for 1 h to obtain a homogenous solution. Then, pure N_2_ was injected into the flask to remove the air until the end of the synthesis. After that, 50 mL 20 mg L^−1^ NaBH_4_ solution at the separating funnel was added dropwise for the reduction of Fe^2+^ to nZVI, being stirred vigorously for 1 h. A leachate separator was used to separate the nZVI/BC particles from the solution and washed several times with ethanol and deionized water in the nitrogen atmosphere. Lastly, clean nZVI/BC nanocomposite was dried in the oven at 60 °C, also under the protection of nitrogen. Two other kinds of nZVI/BC composites with different mass ratios at 1:2 and 1:3 were also prepared based on the nZVI/BC mass ratio of 1:1 procedure, through using 5.0 g FeSO_4_·7H_2_O: 2.0 g BC and 5.0 g FeSO_4_·7H_2_O: 3.0 g BC, respectively. The three types of nZVI/BC with different mass ratios (1:1, 1:2, and 1:3) were labeled as nZVI/1BC, nZVI/2BC, and nZVI/3BC, respectively.

#### 2.2.2. Characterizations

The Brunauer-Emmett-Teller (BET) surface areas and pore size distribution of these samples were measured by nitrogen adsorption (ASAP 2020, Micrometrics, Norcross, GA, USA). The scanning electron microscopy (SEM, QUANTA FEG 250, FEI, Hillsboro, OR, USA), equipped with energy dispersive X-ray spectrometry, was used to observe the morphology and structure of the nZVI, BC, and nZVI/BC at an accelerating voltage of 5 kV. The possible crystalline structures and compositions of these samples were examined through the X-ray diffraction (XRD, XRD-6000, Shimadzu, Japan) test with the 2θ range at 5°–75°. Functional BC groups were characterized by Fourier transform infrared spectroscopy (FT-IR, Nexus 870, Nicolet, WI, USA) spectra, with the wave numbers ranging from 400 to 4000 cm^−1^.

#### 2.2.3. DTZ Adsorption and Degradation

Before degradation, 0.45g L^−1^ peanut shell BC, nZVI/1BC, nZVI/2BC, and nZVI/3BC were added into the 200 mL test solutions prepared with deionized water (20 mg L^−1^ DTZ) in 250 mL conical flasks at 25 °C with an automatic rotation of 125 rpm, respectively, to examine the adsorption of DTZ separately. After no significant difference in adsorption capacity was found, batch experiments of DTZ degradation were performed by adding PS under the previous conditions.

To assess the degradation ability of DTZ, the predetermined amount of activators (nZVI, BC, nZVI/BC) with 25 mM PS were used. To determine the effect of the mass ratio on DTZ degradation, nZVI/BC with different ratios of 1:1, 1:2, and 1:3 were studied. To investigate the impact of the nZVI/BC and PS dosage, 0.15 g L^−1^, 0.30 g L^−1^, 0.45 g L^−1^, and 0.60 g L^−1^ nZVI/BC and 5 mM, 15 mM, 25 mM, and 50 mM PS were used, respectively. To investigate the effect of pH on DTZ degradation, pH values of aqueous solutions were adjusted to 3.0, 5.0, 7.0, 9.0, and 11.0, with 0.1 M sodium hydroxide (NaOH) or 0.1 M sulfuric acid (H_2_SO_4_). To explore the effect of temperature on the oxidation of DTZ in the nZVI/BC-PS system, the temperature was regulated at 15 °C, 25 °C, 35 °C, and 45 °C, respectively. Reusability experiments for the activator (nZVI/2BC) were performed for five cycles under the optimized test conditions. After each recycle, the composite material was filtered and washed with deionized water several times and then cleaned with ethanol before being dried. At selected time intervals, a 800 μL sample was withdrawn and filtered through 0.22 μm syringe filters and immediately put into liquid vials with 200 μL sodium thiosulfate to inhibit reactions prior to analysis.

#### 2.2.4. Analytical Methods

The concentrations of DTZ were determined by a 1200 high-performance liquid chromatograph (HPLC, 1200 series, Agilent, Santa Clara, CA, USA) equipped with a UV detector at 237 nm. The HPLC column used was a reversed-phase C18 column, and the temperature of the column was maintained at 30 °C. The mobile phase was methanol water (20:80, *v*/*v*) with a flow rate at 1 mL min^−1^ [8]. Fe^2+^ produced by the reaction was determined using a SHIMADZU UV-1800 spectroscopy at 510 nm by using the 1,10-phenanthroline colorimetric method, and Fe^3+^ was tested by the reduction with ascorbic acid [45]. PS concentration was analyzed by UV–vis spectrophotometer with KI [46]. The mineralization of DTZ was measured by total organic carbon, using the TOC (Total organic carbon) analyzer (TOC-5000, Shimadzu, Japan).

## 3. Results and Discussions

### 3.1. Characterization of nZVI/BC Composite

**Figure 1:**
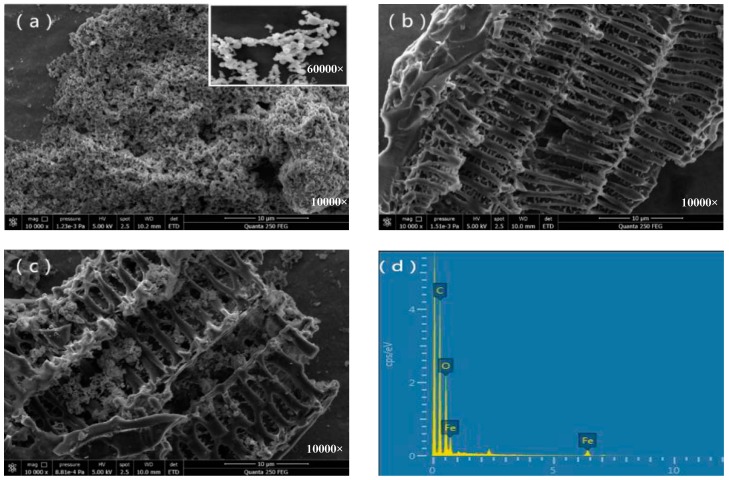
Scanning electron microscopy (SEM) images of nano zero-valent iron (nZVI) (**a**), peanut shell biochar (BC) (**b**), nZVI/peanut shell BC (**c**), and the EDS (Energy-disperse spectra) pattern of nZVI/peanut shell BC (**d**).

With the assistance of SEM, the morphologies of nZVI, BC, and nZVI/BC were clearly observed at a magnification of 10,000 (Figure 1). As seen in Figure 1a, the morphology of nZVI was spherical with a nanoscale particle size, and nZVI particles were easily stacked together due to the magnetic interaction and nano-material properties. The morphology of peanut shell BC was reticular, which showed a skeleton under SEM observation in Figure 1b. The BC structure was well-preserved during the pyrolysis under 350 °C, shown by how the branches of BC are neatly arranged and how each has enough space for nZVI adhesion. Because of the good dispersion of BC, nZVI was evenly distributed on the sticks of the peanut shells (Figure 1c). Therefore, BC made from peanut shells has a unique morphological structure, which could succeed in avoiding the aggregation of nZVI and might furthermore exhibit other good prospects. The EDS (Energy-disperse spectra) elemental mapping can identify the surface element composition of nZVI/BC. In Figure 1d, the spectrum of EDS depicts that the nanocomposite catalyst contains C, O, and Fe. The characterizations above have visually explained the successful synthesis of the nZVI/BC nanocomposite material.

**Figure 2:**
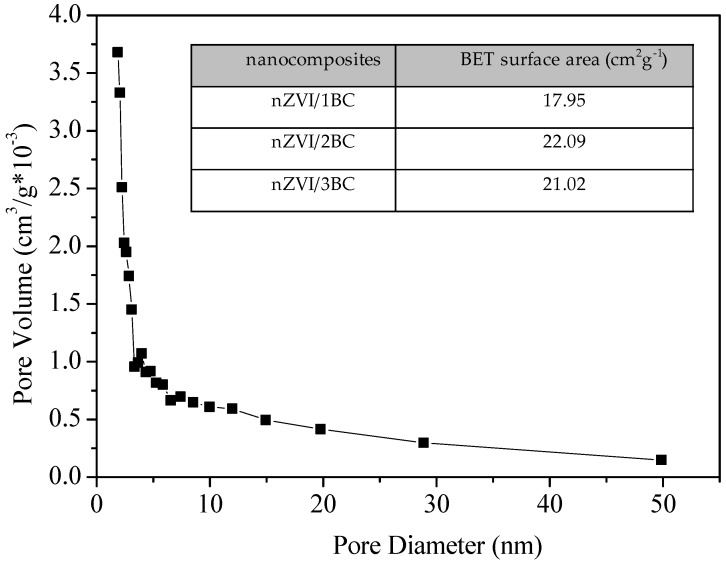
The pore size distribution of nZVI/BC, and the Brunauer-Emmett-Teller (BET) surface area (inset).

The pore size of nZVI/BC was mainly distributed within a range of 2~50 nm, which proved the mesoporous structure of nZVI/BC (Figure 2). The BET surface areas of these materials showed that nZVI/2BC obtained the highest surface area because higher BC content in nZVI/BC composite is beneficial to nZVI dispersion. However, the surface area of nZVI/3BC was decreased by further adding BC contents, owing to the increasing aggregation of BC sheets.

**Figure 3:**
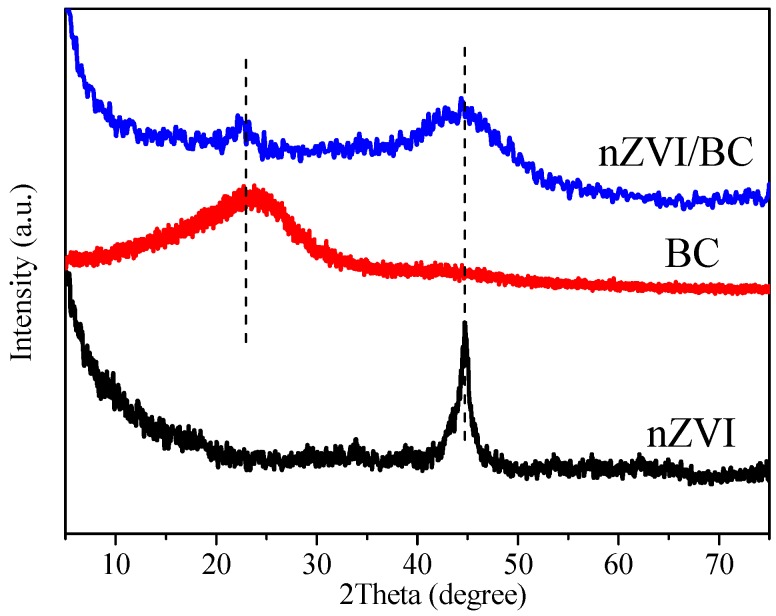
X-ray diffraction (XRD) patterns of nZVI, BC, and nZVI/BC.

The crystalline phases of the prepared samples were determined by powder XRD measurements. The XRD patterns of nZVI, BC, and nZVI/BC are depicted in Figure 3. The diffraction peaks of all samples are shown in the representative position. The peak at 2θ = 45° confirms the presence of α-Fe, which shows that nZVI was obviously synthesized by the liquid-phase reduction method. The representative peak at 2θ = 20°–25° is ascribed to the graphite structure of BC [44]. After synthesis, the characteristics of nZVI and BC are reflected in the particular position of the nZVI/BC diffraction pattern.

**Figure 4:**
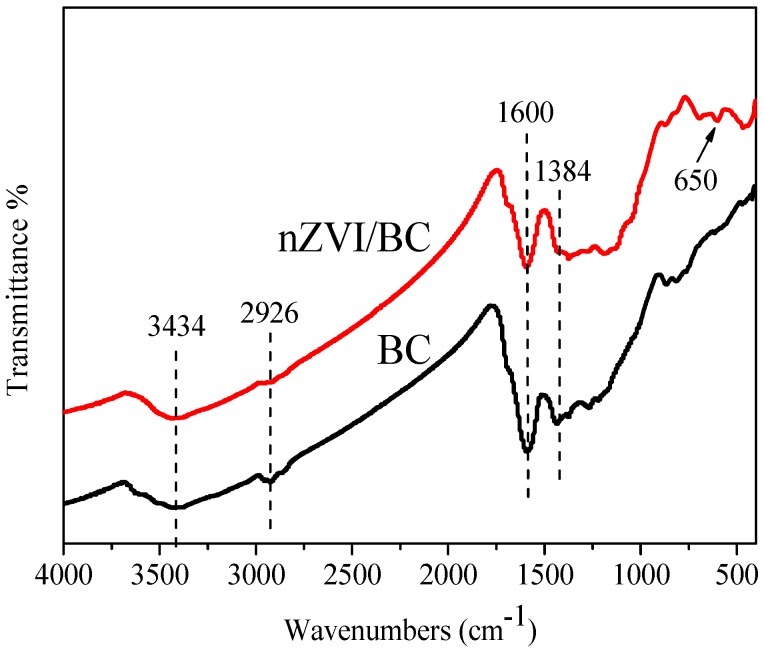
Fourier transform infrared spectroscopy (FT-IR) spectra of BC and nZVI/BC.

FTIR spectra of BC and nZVI/BC are shown in Figure 4. The spectra of nZVI/BC and BC exhibit a broad band at 3434 cm^−1^, which is associated to -OH groups [47]. The band at 1600 cm^−1^ is associated with the carbonyl of the carboxylic group (C=O) [47,48]. The weak peak at 2936 cm^−1^ and 1384 cm^−1^ can be assigned to the bending vibration of C-H [49]. The bonding effects resulting from nZVI and BC might be represented by a peak at 650 cm^−1^ on behalf of Fe-O-H [50]. These characteristic peaks are again proof of the successful preparation of the composite material.

### 3.2. nZVI/BC Activation of PS for DTZ Removal

#### 3.2.1. Adsorption

**Figure 5:**
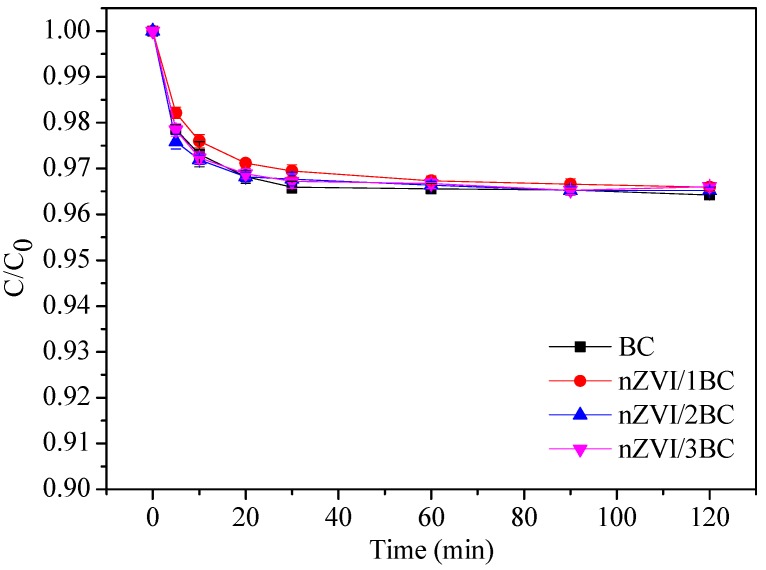
The adsorption of diatrizoate (DTZ) by BC, nZVI/1BC (mass ratio of 1:1), nZVI/2BC (mass ratio of 1:2), and nZVI/3BC (mass ratio of 1:3).

Before the degradation experiment of DTZ, adsorption of the as-prepared material to the target compound was conducted. BC, nZVI/1BC, nZVI/2BC, and nZVI/3BC were used to investigate the effects of adsorption. The adsorption capacity of these materials are shown in Figure 5, where the adsorption removal of DTZ by various materials were only ~3.5% after 120 min, which indicates that adsorption effect of these materials is very low. As can be seen in Figure 5, the adsorption removal rates have no significant difference from 60 min to 120 min, with the rates being ~3.3% to ~3.5%. When considering the time it takes to conduct the experiment, the degradation experiment can be performed after 60 min of adsorption.

#### 3.2.2. Degradation

**Figure 6:**
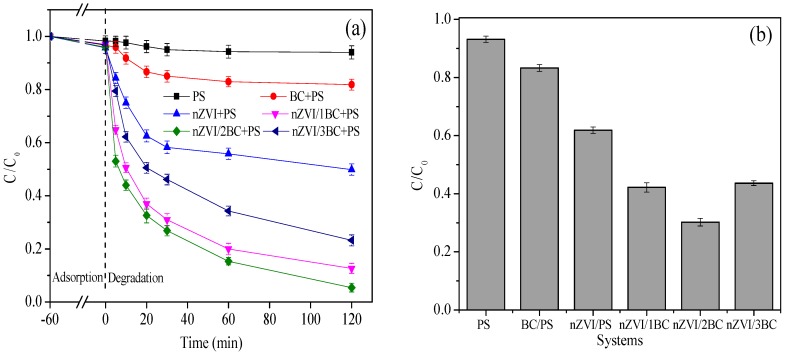
(**a**) The degradation of DTZ in different systems. (**b**) The residual of persulfate (PS) in different systems. [DTZ] = 20 mg L^−1^; [PS] = 25.0 mM; [BC] = 0.30 g L^−1^; [nZVI] = 0.15 g L^−1^; Temp = 25 °C; pH = 7.0.

To highlight the catalytic effect of composite materials in removing DTZ, nZVI, BC, and nZVI/BC composites with different mass ratios (1:1–1:3) in the presence of PS were investigated for 120 min after being largely unaffected by adsorption (60 min). The entire experiment lasted 180 min. Figure 6a shows that PS alone was the least effective (~6%) at DTZ removal. This is because PS has weak oxidation ability (E_0_ = 2.01 V) due to its low activation and few sulfate radicals generated. The sample BC-PS exhibited better performance than PS alone, with the DTZ degradation rate being 14.7%. This could be due to the fact that the PS was slightly activated by the function groups in the BC mentioned in the infrared characterization [50,51,52]. The ability of nZVI in activating PS was confirmed by its 45.8% DTZ removal rate. Meanwhile, the consumption of PS by nZVI-PS was ~38% (Figure 6b), which demonstrated that activation by nZVI alone could not fully develop the potential of PS. Although there was no significant difference in the adsorption capacities of the three nanocomposites with various mass ratios, the ability to activate persulfate varied greatly. The DTZ degradation rates by PS activated with nZVI/1BC, nZVI/2BC, and nZVI/3BC were 84.3%, 91.5%, and 73.6% respectively after deducting the quantity of adsorption. Meanwhile, the remaining PS concentration ratios were at 42.2%, 30.2%, and 44.6%, respectively. nZVI/2BC exhibited the best catalytic capability in the degradation of DTZ, and the utilization of PS was the highest. The removal of DTZ by nZVI/1BC was inferior to nZVI/2BC, which might be due to the fact that there was not enough BC to disperse the large amount of nZVI. However, excessive BC may also have a negative impact on the generation of SO_4_^•−^ through reducing the contact between nZVI and PS, resulting from the aggregation of BC sheets [44,49]. Therefore, the nZVI/2BC was identified to be the most effective composite material and was selected as the activation reagent for further study.

### 3.3. Effect of nZVI/2BC and PS Dosage

**Figure 7:**
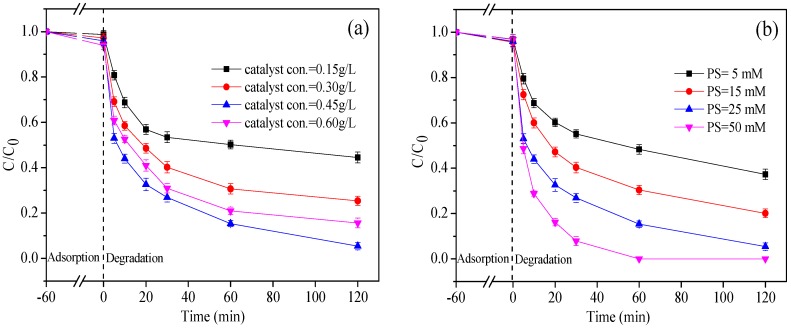
(**a**) Effect of nZVI/2BC dosages on the degradation of DTZ; (**b**) Effect of PS concentrations on the degradation of DTZ. [DTZ] = 20 mg L^−1^; Temp = 25 °C; pH = 7.0; [PS] = 25.0 mM for (**a**) or [nZVI/2BC] = 0.45 g L^−1^ for (**b**).

Figure 7 shows the effect of nZVI/2BC and PS dosages on the degradation of DTZ. In Figure 7a, the DTZ removal rates ranges from 54.2% to 91.5% under dosages of nZVI/2BC ranging from 0.15 to 0.45 g L^−1^ with 25 mM PS. The trend shows that the degradation efficiency increases with the amount of nZVI/2BC being applied. Because the loaded nZVI is accompanied by an increase in the amount of composite materials, Fe^2+^ would be persistently released to activate PS to generate more SO_4_^•−^, correspondingly. However, the degradation efficiency using 0.60 g L^−1^ nZVI/2BC is shown to decrease to 78.3%, which may be due to how excessive Fe^2+^ can integrate with SO_4_^•−^. Thus, some of SO_4_^•−^ did not participate in the degradation of DTZ but reacted with Fe^2+^ as a scavenging reaction (Equation (1)).


SO_4_^•−^ + Fe^2+^ → S_2_O_8_^2−^ + Fe^3+^ (1)

The effects of the different PS concentration levels (5, 15, 25, and 50 mM) with 0.45 g L^−1^ nZVI/2BC on the degradation of DTZ is shown in Figure 7b. The removal rates of DTZ in four groups were 59.6%, 75.4%, 91.5%, and 100%, respectively. It can be seen that the higher the concentration of PS, the better the degradation of DTZ. In addition, DTZ degrades rapidly in the first 20 min, and 50 mM PS was able to fully degrade the DTZ activated by nZVI/2BC in 120 min. With the increase in PS dosage, plenty of SO_4_^•−^ were generated due to the intensive decomposition of PS, which enhanced the degradation of DTZ.

### 3.4. Effect of Reaction Temperature and Solution pH

It was reported that heat could activate PS to release sulfate radicals by opening the O–O bond [53,54] (Equation (2)).


S_2_O_8_^2−^ + heat → 2SO_4_^•−^ (2)

**Figure 8:**
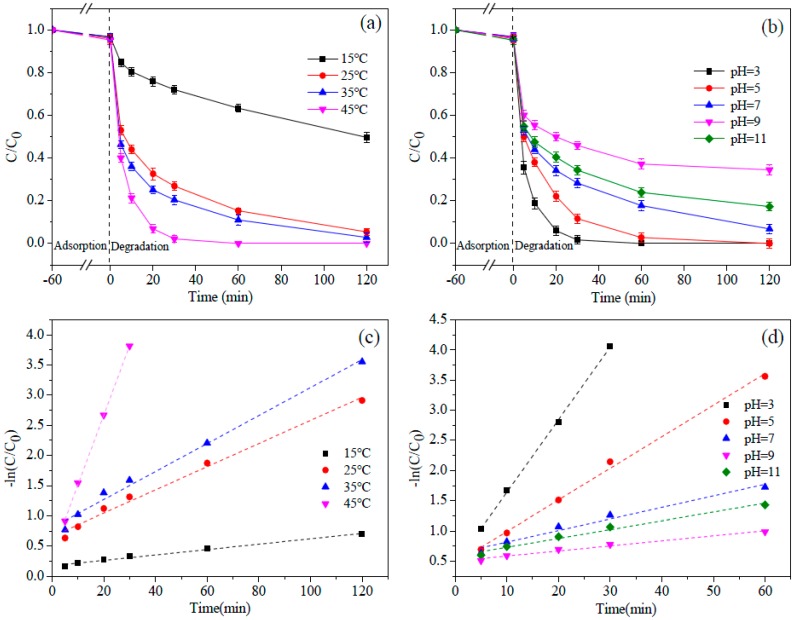
(**a**) Effect of temperature on the degradation of DTZ. [DTZ] = 20 mg L^−1^; [nZVI/2BC] = 0.45 g L^−1^; [PS] = 25.0 mM; pH = 7.0. (**b**) Effect of pH on the degradation of DTZ. (**c**) Corresponding kinetic curves of DTZ with different temperatures. (**d**) Corresponding kinetic curves of DTZ with different pH values [DTZ] = 20 mg L^−1^; [nZVI/2BC] = 0.45 g L^−1^; [PS] = 25.0 mM; Temp = 25 °C.

In this study, the effect of the reaction temperature on the activation of PS with nZVI/2BC was studied. It can be seen from Figure 8a that the low temperature significantly restricted the degradation efficiency of DTZ. More specifically, when the temperature was 15 °C, the degradation efficiency of DTZ was only 47.1% after 120 min, and when the temperature was 25 °C, the degradation rate greatly increased to ~92%. It can thus be inferred that the degradation reaction is very sensitive to temperature, and is most affected between 15 °C and 25 °C. When the nZVI/2BC-PS system performed at 45 °C, the removal rate of DTZ was nearly 100% in 30 min. The results also imply that DTZ degradation follows the pseudo-first-order kinetic equation (Figure 8c). The fitting coefficients *R*^2^ were over 0.99, and DTZ reaction kinetics rate constants were equal to 0.0045, 0.0218, 0.0268, and 0.1232 min^−1^ at 15, 25, 35, and 45 °C, respectively (Table 1). According to the laws of thermodynamics and the experimental results, the reaction kinetics rate constant *k* increased as the temperature increased, and both of them fit with the Arrhenius equation (Equation (3)).

(3) $$K=A\exp(\frac{-Ea}{RT})$$

A straight linear correlation between ln*K* and 1/T was achieved. The calculated *Ea* was approximately 9.07 × 10^3^ J mol^−1^ within the range of 15–45 °C, and the pre-exponential factor *A* was 26.17 min^−1^, showing that the reaction was endothermal.

It is well-known that solution pH is one of the most important influencing factors which affect the degradation of DTZ by the nZVI/BC-PS system. The effect of solution pH on the degradation of DTZ was investigated. As shown in Figure 8b, the order of DTZ removal is pH 3.0 > pH 5.0 > pH 7.0 > pH 11.0 > pH 9.0. The maximum removal was 100% at pH 3.0, and the minimum removal rate was 61.8% at pH 9.0. The degradation of DTZ increased as the solution pH decreased from 9.0 to 3.0. This tendency can be explained as follows (Equations (4) and (5)): Under acidic conditions, sulfate radicals are easily motivated because of acid catalysis [11,55], which is consistent with the previous study which found that acidic pH (pH 3.0) favored the removal of nonylphenol and trichloroethylene [44,49]. Under basic conditions, some sulfate radicals can react with hydroxyl or H_2_O. A small quantity of generated hydroxyl radicals had relatively shorter lifetimes compared to sulfate radicals, which had a serious impact on the degradation of organic pollutants.


S_2_O_8_^2−^ + H^+^ → HS_2_O_8_^−^ (4)

HS_2_O_8_^−^ → H^+^ + SO_4_^2−^ + SO_4_^•−^ (5)

SO_4_^•−^ + OH^−^ → HO^•^ + SO_4_^2−^ (6)

However, the degradation rate of DTZ was 78.6% at pH 11, which is higher than that at pH 9.0. Under extremely alkaline conditions, the sulfate radical produced can catch an electron from hydroxyl, forming a predominate hydroxyl radical that also has high redox potential [56,57,58] (Equation (6)). Basic activation caused by the solution of pH 11 was applicable to the degradation of DTZ. By calculating the kinetic equation, the highest DTZ degradation rate of pH 3 (0.1294 min^−1^) was ~2.5, ~7.5, ~15.5, and ~8.9 times faster than those of pH 5 (0.0523 min^−1^), pH 7 (0.0171 min^−1^), pH 9 (0.0084 min^−1^), and pH 11 (0.0146 min^−1^). Therefore, the nZVI/2BC-PS system can effectively degrade refractory organic pollutants when under an acidic condition followed by a strong alkaline condition.

### 3.5. DTZ Mineralization

**Figure 9:**
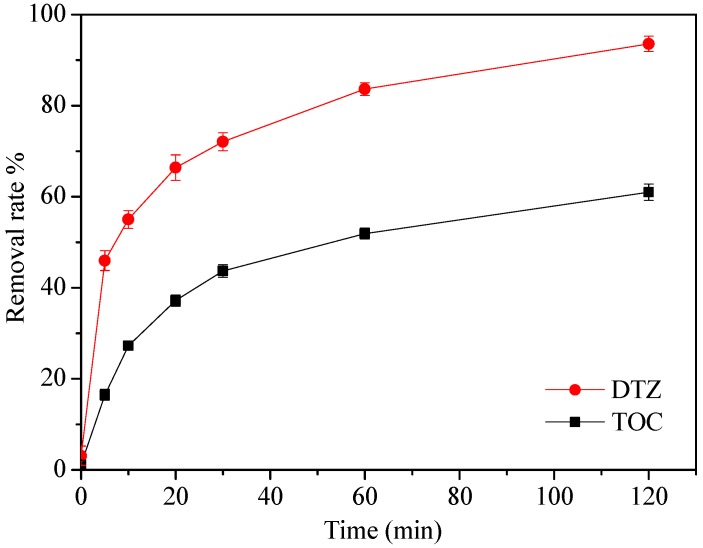
The removal rate of TOC (Total organic carbon) and DTZ. [DTZ] = 20 mg L^−1^; [nZVI/2BC] = 0.45 g L^−1^; [PS] = 25.0 mM; Temp = 25 °C; pH = 7.0.

It is necessary to analyze the concentrations of TOC in each aqueous solution, since the purpose of the DTZ degradation was not only the degradation itself but also to obtain its mineralization. In Figure 9, the trend line of the mineralization is roughly similar to the removal rate of DTZ, and the ultimate mineralization of DTZ is 61% after 120 min, which is reasonable and shows that decomposition was not fully completed. The intermediate products were produced and required a longer time to mineralize them thoroughly.

### 3.6. Activation Mechanism

**Figure 10:**
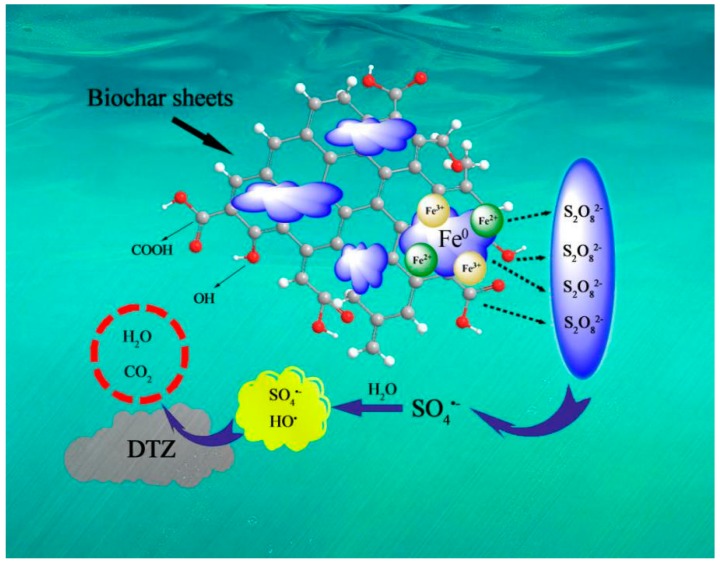
The mechanism of nZVI/BC activating PS for the degradation of DTZ. [DTZ] = 20 mg L^−1^; [nZVI/2BC] = 0.45 g L^−1^; [PS] = 25.0 mM; Temp = 25 °C; pH = 7.0.

The activation mechanism of PS is generally described in Figure 10. Peanut shell BC played a very important role in the degradation of DTZ. The degradation efficiency of DTZ (14.7%) in the BC-PS system was higher than the DTZ removal rate (~6%) with PS alone. It showed that peanut shell BC was able to activate PS to release SO_4_^•−^ based on the abundant oxygen functional groups (containing BC-COOH and BC-OH) on the BC surface (Equations (7) and (8)) [59,60]. In addition, the well-arranged mesh structure of peanut shell BC provided enough binding sites for nZVI, which was beneficial in dispersing the nZVI magnetic particles for full activation.


BC-OOH + S_2_O_8_^2−^ → BC-OO^•^ + SO_4_^•−^ + HSO_4_^−^ (7)

BC-OH + S_2_O_8_^2−^ → BC-O^•^ + SO_4_^•−^ + HSO_4_^−^ (8)

**Figure 11:**
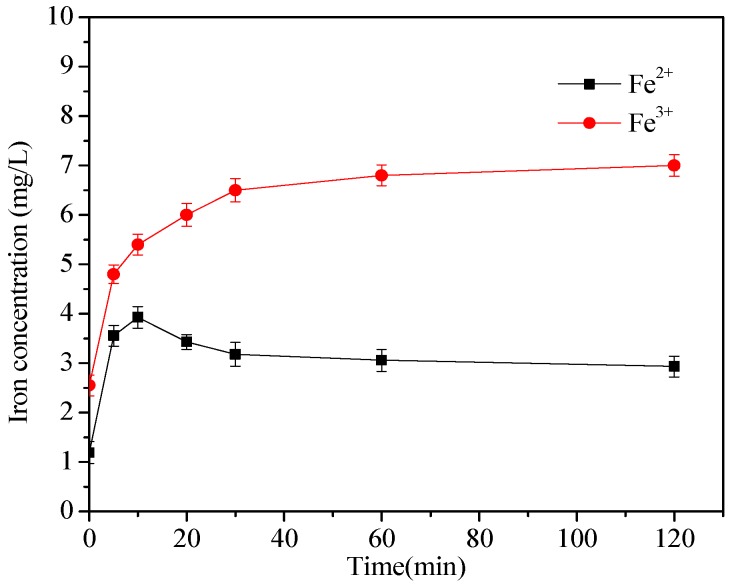
Concentration of Fe^2+^ and Fe^3+^ in the nZVI/2BC-PS system. [DTZ] = 20 mg L^−1^; [nZVI/2BC] = 0.45 g L^−1^; [PS] = 25.0 mM; Temp = 25 °C; pH = 7.0.

Figure 11 shows the concentration of dissolved iron and the conversion among Fe^0^, Fe^2+^, and Fe^3+^. The concentrations of Fe^2+^ and Fe^3+^ increases in the first five minutes, and the concentration of Fe^2+^ slowly declines after reaching the peak around 10 min, after which the concentration of Fe^3+^ maintains a slow rise until it levels off. After 120 mins of reaction time, the concentrations of Fe^2+^ and Fe^3+^ were 2.93 mg L^−1^ and 7.02 mg L^−1^, respectively. The results indicate that a part of nZVI was transformed into dissolved iron in the presence of PS: Fe^0^ activated PS to form SO_4_^•−^, HO^•^, and Fe^2+^ in the aqueous solutions (Equations (9)–(11)). Meanwhile, the formed Fe^2+^ could also react with S_2_O_8_^2−^ to generate SO_4_^•−^ and Fe^3+^ (Equation (11). The excessive SO_4_^•−^ might react with Fe^2+^ to form Fe^3+^ (Equation (1)). Therefore, the concentration of Fe^2+^ increased first, then went down to equilibrium, and finally, the concentration of Fe^3+^ was higher than Fe^2+^.

Fe^0^ + 2S_2_O_8_^2−^ → Fe^2+^ + 2SO_4_^2−^ + 2SO_4_^•−^ (9)

Fe^0^ + S_2_O_8_^2−^ + 2H_2_O → 2SO_4_^2−^ + Fe^2+^ + 2HO^•^ + 2H^+^ (10)

Fe^2+^ + S_2_O_8_^2−^ → Fe^3+^ + SO_4_^2−^ + SO_4_^•−^ (11)

**Figure 12:**
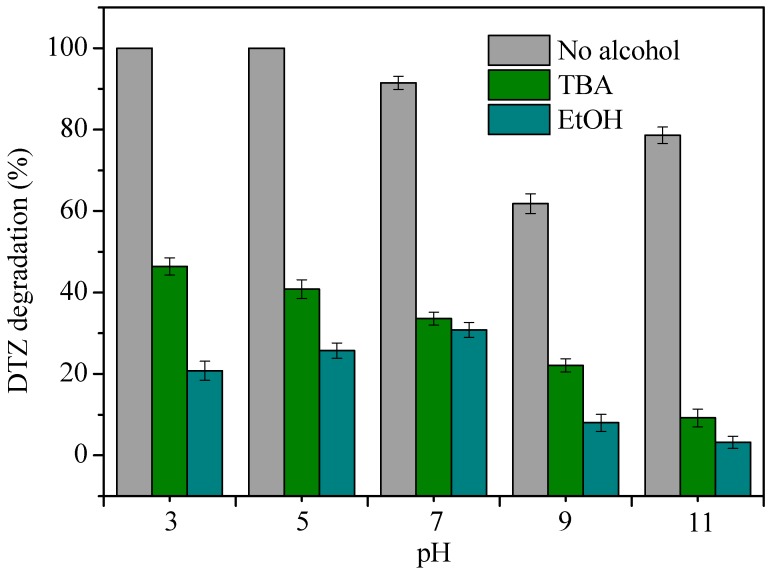
Effects of tert-butyl alcohol (TBA) and ethanol (EtOH) on the degradation of DTZ. [DTZ] = 20 mg L^−1^; [nZVI/2BC] = 0.45 g L^−1^; [PS] = 25.0 mM; Temp = 25 °C; pH = 7.0.

In order to figure out the radical species responsible for DTZ oxidation, sulfate radical and hydroxyl radical were investigated in comparative experiments at different pH values at 25 °C. Tert-butyl alcohol (TBA) and ethanol (EtOH) were the probe reagents used to examine the main radical species for the degradation of DTZ, as they had different quenching functions. The quenching effects of TBA to HO^•^ are stronger than that of SO_4_^•−^ (K_SO4_^•−^ = 4.0–9.1 × 10^5^ M^−1^s^−1^, K_HO_^•^ = 3.8–7.6 × 10^8^ M^−1^s^−1^). Both SO_4_^•−^ and HO^•^ can be effectively quenched by EtOH and the reaction rate constants of EtOH with SO_4_^•−^ and HO^•^ are 1.6–7.7 × 10^7^ M^−1^s^−1^ and 1.2–2.8 × 10^9^ M^−1^s^−1^, respectively [44,61]. Therefore, 0.1M TBA and 0.1M EtOH were taken into the DTZ degradation system to determine the main radical species. The differences in degradation with or without the quenching reagent can be seen clearly in Figure 12.

When pH ranges from 3.0 to 7.0, the blank sample exhibits favorable degradation. However, the degradation process was significantly inhibited, with removal efficiency decreasing from 100% to 46.4% and 100% to 20.8% by adding TBA and EtOH at pH 3.0, respectively. The inhibition from EtOH was stronger than TBA, indicating that SO_4_^•−^ played a dominant role at acidic and neutral conditions. When the pH value was around 11.0, the degradation of DTZ was 9.2% for TBA-quenching and 3.2% for EtOH-quenching, respectively. Both groups were significantly inhibited, especially for the TBA-quenching sample, which implies the main radicals in alkaline solution were HO^•^. The quenching experiments and the above discussion for influencing factors fully demonstrate that pH plays a very important role on the degradation of DTZ by the nZVI/BC-PS system.

### 3.7. The Stability of the nZVI/BC

**Figure 13:**
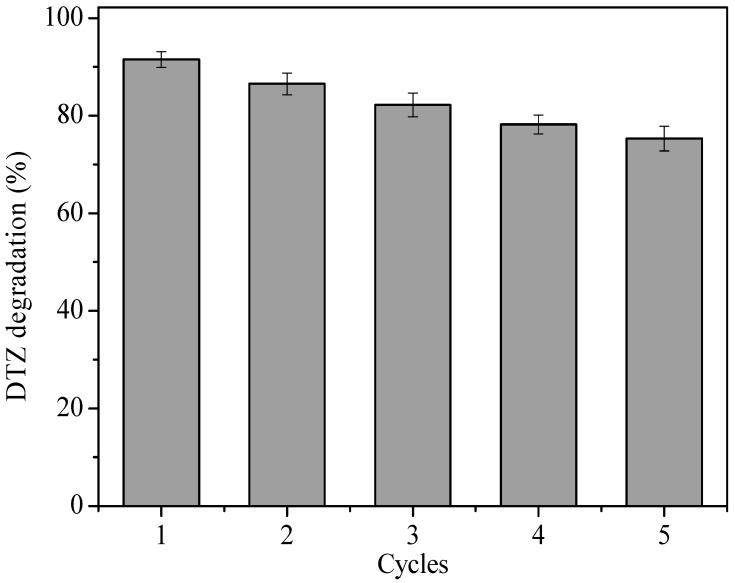
Reusability investigation of nZVI/2BC composite materials for DTZ degradation. [DTZ] = 20 mg L^−1^; [PS] = 25.0 mM; [nZVI/2BC] = 0.45 g L^−1^; Temp = 25 °C; pH = 7.0.

Compared to simply adding nZVI, nZVI/BC was easier to collect after the experiment and could be recycled multiple times by peanut shell BC adhesion. As can be seen from the histogram (Figure 13), the five degradation rates of DTZ were 91.5%, 86.5%, 82.2%, 77.2%, and 75.3%, in turn. As the recycle increased, the degradation of DTZ decreased slowly. The degradation rate reduction might owe to: (1) The adsorption of DTZ on composite materials inhibiting the activation of PS by the catalyst; (2) Fe^2+^ running away led to the decrease of binding sites on the nZVI/2BC surface; (3) nZVI loaded on BC might have come off due to multiple flushing. Nevertheless, the nZVI/BC-PS can still degrade 77.3% of DTZ at the fifth run, which shows its potential in applicability.

## 4. Conclusions

nZVI/BC (peanut shell) nanocomposite activating PS exhibited good efficiency for the degradation of DTZ. The results demonstrated that: (1) nZVI/2BC is an efficient nanocomposite, where the DTZ removal rate in the experiment (pH 7) was 94.6% (91.5% degradation and 3.1% adsorption), and the DTZ removal rate in the optimized experiment (pH 3.0) was nearly 100%. (2) The degradation efficiency of DTZ was affected by the dosage of nZVI/BC and PS, reaction temperature, and solution pH. (3) nZVI on the surface of peanut shell BC enhanced the redox effect between Fe^2+^ and Fe^3+^, which promoted the generation of SO_4_^•−^. (4) Free radical quenching experiments revealed that both SO_4_^•−^ and HO^•^ were responsible for the degradation of DTZ. (5) Nanocomposite nZVI/BC prepared by peanut shell was recycled five times and considered to be an efficient catalyst of PS for the treatment of refractory organic contaminants.

## Figures and Tables

**Table 1:** The kinetic parameters of DTZ removal by different temperatures and pH.

Factors	Gradient	Kinetic Equations	*R* ^2^	*k*/(min^−1^)
Temperature	15 °C	−ln(*C*/*C*_0_) = 0.0045 t + 0.1745	0.9912	0.0045
	25 °C	−ln(*C*/*C*_0_) = 0.0192 t + 0.6622	0.9903	0.0192
	35 °C	−ln(*C*/*C*_0_) = 0.0232 t + 0.8061	0.9912	0.0232
	45 °C	−ln(*C*/*C*_0_) = 0.1154 t + 0.3617	0.9997	0.1154
pH	3	−ln(*C*/*C*_0_) = 0.1294 t + 0.2391	0.9912	0.1294
	5	−ln(*C*/*C*_0_) = 0.0523 t + 0.4676	0.9970	0.0523
	7	−ln(*C*/*C*_0_) = 0.0171 t + 0.6687	0.9901	0.0171
	9	−ln(*C*/*C*_0_) = 0.0084 t + 0.5021	0.9808	0.0084
	11	−ln(*C*/*C*_0_) = 0.0146 t + 0.5854	0.9822	0.0146
